# Order parameter analysis for low-dimensional behaviors of coupled phase-oscillators

**DOI:** 10.1038/srep30184

**Published:** 2016-07-22

**Authors:** Jian Gao, Can Xu, Yuting Sun, Zhigang Zheng

**Affiliations:** 1 College of Information Science and Engineering, Huaqiao University, Xiamen 361021, China; 2 Department of Physics and the Beijing-Hong Kong-Singapore Joint Center for Nonlinear and Complex Systems (Beijing), Beijing Normal University, Beijing 100875, China

## Abstract

Coupled phase-oscillators are important models related to synchronization. Recently, Ott-Antonsen(OA) ansatz is developed and used to get low-dimensional collective behaviors in coupled oscillator systems. In this paper, we develop a simple and concise approach based on equations of order parameters, namely, order parameter analysis, with which we point out that OA ansatz is rooted in the dynamical symmetry of order parameters. With our approach the scope of OA ansatz is identified as two conditions, i.e., the limit of infinitely many oscillators and only three nonzero Fourier coefficients of the coupling function. Coinciding with each of the conditions, a distinctive system out of the scope is taken into account and discussed with the order parameter analysis. Two approximation methods are introduced respectively, namely the expectation assumption and the dominating-term assumption.

Understanding the intrinsic mechanism of collective behaviors of coupled units has become a focus for a variety of fields, such as biological neurons, circadian rhythm, chemically reacting cells, and even social systems^1^,^2^,^3^,^4^,^5^,^6^,^7^. Some properties of collective behaviors depend on complexity of the system, while the other properties, such as phase transitions, may be described by low-dimensional dynamics macroscopic variables^8^,^9^,^10^,^11^,^12^. Discovering methods to simplify the system is as important and as fascinating as the discovery of complexity of it.

Like most cases in physics, simplification and low-dimensional reduction are associated with some symmetry of the system. As the identity of gas particles is the foundation of statistical mechanics and collective variables as temperature and pressure, the identity of coupled units in a complex system is also related to some order parameters. In previous works, in the limit of infinite many oscillators *N* → ∞, this work has been done through Ott-Antonsen(OA) ansatz^12^ for nonidentical oscillators. The same result for identical oscillators was also got from group theory analysis in refs 10 and 13, called Watanabe-Strogatz’s approach.

In this paper, we will show a different way to get the low-dimensional reduction, where it is a natural consequence of the symmetry of the system by taking order parameters as collective variables, namely the order parameter analysis. Our approach is simple and concise for identical and nonidentical oscillators. We can also get the scope of OA ansatz, with two more cases beyond the scope discussed further with appropriate approximations. With our approach we show that OA ansatz can be used beyond its scope with some approximations.

The model discussed in this paper is all-connected coupled phase-oscillators. In the first section, dynamical equations of order parameters are derived. OA ansatz is got naturally from the symmetry of the order parameter equations, with its scope as the limit of infinitely many oscillators and the condition that only three Fourier coefficients of the coupling function are nonzero. In the second and third sections, we will consider two approximate use of this ansatz beyond its scope, finite-size systems and coupled oscillator systems with more complicated coupling functions, with approximations respectively, i.e., the expectation assumption and the dominating-term assumption.

## Results

### Equations for order parameters

The famous Kuramoto model for the process of synchronization has been attracting many attentions upon it is proposed and has been developed for decades. This model consists of a population of N coupled phase oscillators {*φ*_*j*_} with natural frequencies {*ω*_*j*_}, and the dynamics are described by the following equations of motion:


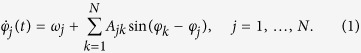


With the mean-field coupling *A*_*jk*_ = *K*/*N* and the definition of an order parameter


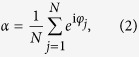


the equation (1) can be rewritten as


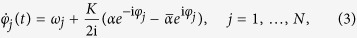


where i is the imaginary unit, 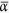 is the complex conjugate of *α*. Apart from parameters *K* and *ω*_*j*_, the dynamics of each phase variable *φ*_*j*_ depends only on itself and the order parameter *α*, which is an important characteristic of this mean-field model. The order parameter *α* can also be used to describe collective states of the system, as the system is in synchronous states if and only if |*α*| = 1.

A more general form of this mean-field phase-oscillator model can be written as


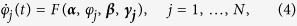


where *F*(***α***, *φ*_*j*_, ***β***, ***γ***_***j***_) is any smooth, real, 2*π*-periodic function of *φ*_*j*_. ***α*** = (…*α*_−1_, *α*_0_, *α*_1_…) are a group of order parameters with the *n*th order parameter *α*_*n*_ defined as


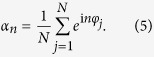


***β*** = (*β*_1_, *β*_2_ … *β*_*s*_) are homogeneous parameters, such as the coupling strength *K*, which are identical for all the oscillators. 
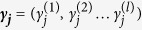
 are a group of inhomogeneous parameters, such as the natural-frequencies {*ω*_*j*_}, which are nonidentical for the oscillators and usually obey certain distributions among the system.

Almost all the mean-field models based on Kuramoto model belong to this general category. In the following, we will build our approach for this general model to explore the conditions under which we can get a low-dimensional description of system (4).

#### Identical oscillators

To begin with, let us consider the simple case of identical oscillators, e.g., *ω*_*j*_ = ω* for *j* = 1, 2, …, *N* in Eq. (4), which reads


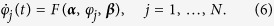


In the limit of infinitely many oscillators *N* → ∞, let *ρ*(*t*, *φ*)*dφ* denote the fraction of oscillators whose phases lie between *φ* and *φ* + *dφ*. According to Eq. (6), each oscillator moves with an angular velocity *v*_*j*_ = *F*(***α***, *φ*_*j*_, ***β***). The single oscillator density *ρ*(*t*, *φ*) obeys the continuity equation as


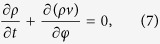


where *v* = *F*(***α***, *φ*, ***β***). If the phase density *ρ*(*t*, *φ*) is known, all of macroscopic properties of the system can be got through the statistical average over *φ*, such as order parameters *α*_*n*_ defined as


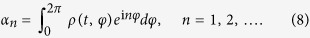


If we are only concerned with collective or macroscopic states of the system, as in this paper, the macroscopic description with a phase density is equivalent to the microscopic description with phases of all the oscillators.

Moreover, as the coupling function *F*(***α***, *φ*, ***β***) is always 2*π*-periodic for *φ*, it can be written as the Fourier expansion,


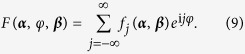


With definitions of order parameters Eq. (8) and the continuity equation Eq. (7), dynamics of order parameters can be got as


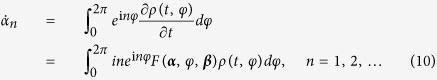


Substituting the expansion of *F*(***α***, *φ*, ***β***) (9) into Eq. (10), we have the dynamical equations of order parameters as


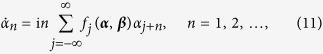


which is equivalent to the continuity equation Eq. (7).

The complexity of equation (11) depends on the coupling function *F*, or explicitly, on the Fourier coefficients {*f*_*j*_(***α***, ***β***)}. First, let us consider the simplest case, where only the first three terms of the Fourier expansion are nonzero,


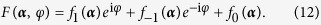


Because *F* is a real function, we have 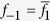, where 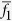 means the complex conjugate of *f*_1_. With the expansion Eq. (12), the dynamics of order parameters Eq. (11) become


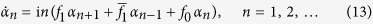


with 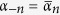.

The recursion form of Eq. (13) shows that there is some structures of order parameters from which we can simplify the system and get low-dimensional equations to describe the system with this symmetry. For the simplest case, supposing *α*_*n*_ = *G*(*α*_1_, *n*), where *G*(*α*_1_, *n*) is a function, we have


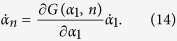


According to Eq. (13), by substituting 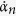 and 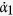 into Eq. (14), due to the independence between *f*_1_, 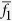 and *f*_0_ we have


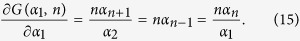


From the third equality of Eq. (15), one gets


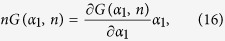


which can be solved as 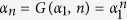. With this relation, the other two equalities in Eq. (15) are satisfied automatically. The relation 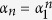, *n* ≥ 0 is the structure of order parameters which represents the symmetry of the system.

As a result, by choosing a manifold as 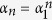, *n* ≥ 0, all the dynamical equations of order parameters Eq. (13) are reduced to a single one,


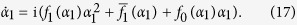


If we choose the initial state in this manifold, the system will never evolve out of it. The manifold 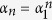, *n* ≥ 0 is invariant under the dynamics Eq. (13). This is exactly the low-dimensional behaviors of the system we are looking for. It is also found that this low-dimensional invariant manifold is stable and attractive in some cases^14^,^15^,^16^.

Corresponding to this manifold 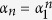, *n* ≥ 0, we have the phase density *ρ*(*φ*) as


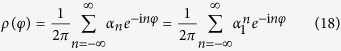


which is the so-called Poisson kernel distribution as


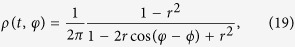


where *α*_1_ = *re*^i*ϕ*^. We will call this manifold Poisson manifold in this paper as in ref. 10 where the dynamic of *α*_1_, as Eq. (17), is got from a group theory analysis of Josephson junction arrays.

#### Nonidentical parameters

Low-dimensional behaviors are not confined to systems of identical oscillators. The order parameter analysis we used above can also be used in more general cases of nonidentical oscillators.

Let us consider a system of nonidentical oscillators with a distribution of the natural frequency *g*(*ω*). Dynamical equations of this mean-field coupled oscillator model read


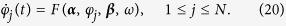


In the limit of infinitely many oscillators *N* → ∞, the fraction of oscillators with natural frequencies between *ω* and *ω* + *dω* and phases between *φ* and *φ* + *dφ* can be defined as *ρ*(*φ*, *ω*)*dφdω*, with which order parameters {*α*_*n*_} read


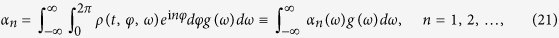


where {*α*_*n*_(*ω*)} are local order parameters.

One can get the continuity equation of the single oscillator density *ρ*(*φ*, *ω*) as


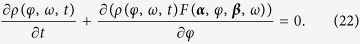


As the coupling function *F*(***α***, *φ*, ***β***, *ω*) is 2*π*-periodic for *φ*, one can expand it with Fourier basis. Together with the definition of local order parameters and the continuity equation, we have


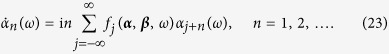


The dynamics are closed for local order parameters {*α*_*n*_(*ω*)}, which can be regarded as coordinates of the system and equivalent to phase variables.

From Eq. (23), each group with different *ω* is almost separated from the others and the only relation between the groups is the dependence on order parameters ***α***. It is obvious that in the simplest case where only the first three terms of Fourier expansions are nonzero, Poisson manifold is invariant for each group, and the system described by {*α*_*n*_(*ω*)} is governed by a group of Poisson manifolds with low-dimensional dynamics.

Up to now, we have discussed the system of all-connected phase oscillators in the limit of infinitely many oscillators and the condition that only three Fourier coefficients of the coupling function are nonzero. The low-dimensional invariant manifold, namely Poisson manifold, is got with these two terms. We will see in the next two sections that these two conditions are exactly the scope of OA ansatz, with two cases beyond this scope discussed respectively.

#### Network with finite size

We have considered the general model Eq. (6) in the limit of infinitely many oscillators, *N* → ∞, where we can get the phase density of oscillators *ρ*(*t*, *φ*) and the corresponding continuity equation (7). Order parameters {*α*_*n*_} can be considered as Fourier coefficients of *ρ*(*t*, *φ*), and we get an equivalent expression of the dynamics of the system as the order parameter equation (11) and build the approach above.

For the system with finite number of oscillators, order parameters {*α*_*n*_} can not be considered as well-defined Fourier coefficients of *ρ*(*t*, *φ*), but it can be considered as collective variables as


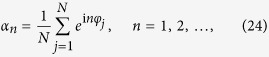


which does not depend on the approximation of infinitely many oscillators.

For the mean-field phase-oscillator system,


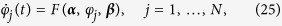


with the definition of order parameters Eq. (24), dynamical equations of order parameters read


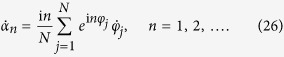


As the coupling function *F*(***α***, *φ*_*j*_, ***β***) is 2*π*-periodic for *φ*_*j*_, one has its Fourier expansion as


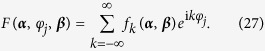


By substituting this expansion into Eq. (26) and combining with Eq. (24), we obtain the dynamical equations of order parameters as


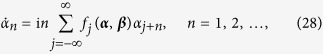


and this expression is the same as that we got in the limit of infinitely many oscillators [see Eq. (11)].

Following our approach, when only the first three Fourier coefficients of the coupling function are nonzero, one can get an invariant manifold with 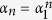. By substituting this relation into dynamical equation (28), one can get a reduced equation as


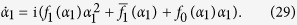


It appears that we could get Poisson manifold again even the size of the system is finite. However, the finite-size effect should be taken into account with care. As a matter of fact, Poisson manifold with 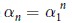 is not attainable for finite systems. To see this, let us take the system with *N* = 2 as an example. For the first two order parameters *α*_1_ and *α*_2_, by definition one has


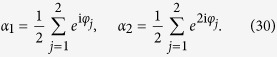


If the system described by *α*_1_, *α*_2_ is in Poisson manifold, it should satisfy the relation 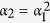 which can be rewritten as


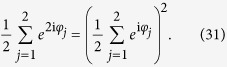


However, the equality Eq. (31) is not naturally valid. By using a simple calculation of the difference between the left-hand and the right-hand terms in Eq. (31) one obtains


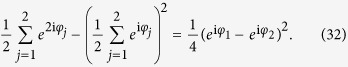


This indicates that the system can evolve on Poisson manifold only when *φ*_2_ = *φ*_1_.

In general, the relation 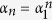 gives exactly infinitely many independent constraints like Eq. (31) with *n* = 2, 3, …. In this manifold, any solutions of finite systems will be determined as the trivial one as *φ*_*j*_ = *φ*_1_ for all the index *j*, which means that except synchronous states, all the other states of finite systems lie out of this Poisson manifold. Systems can only evolve on Poisson manifold in the limit of infinitely many oscillators.

Whereas, as a matter of fact, the equation (28) hold for all *N*, whether finite or infinite. Synchronous states always satisfy the relation of Poisson manifold, which can be used to describe the system at least approximately in the vicinity of synchronous states.

As this Poisson manifold reflects the symmetry of the order parameter equation (28), it is an interesting question that under what conditions this symmetry and the corresponding Poisson manifold governs the behaviors of system.

In general, by choosing initial states of *N* oscillators randomly from a probability density in Poisson manifold as


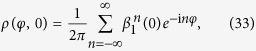


one has the expectation value of *e*^i*nφ*^ as


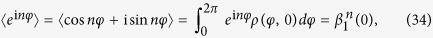


and variances of the real and imagine part of *e*^i*nφ*^ as


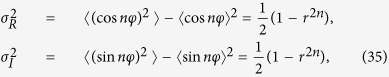


where *r* = |*β*_1_(0)|. Hence expectations and variances of initial order parameters 
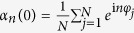
 of the system with *N* oscillators read


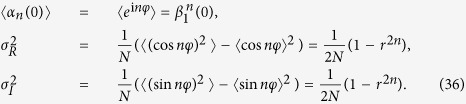


If the initial expectations satisfy the relation 〈*α*_*n*_(0)〉 = 〈*α*_1_(0)〉^*n*^, and the variances will vanish in the limit *N* → ∞ or *r* → 1. This clearly indicates that when the size of the system is large enough 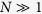 or in the vicinity of synchrony *r* ≈ 1, the order parameter equations can be used to describe the behaviors of the system approximately. The symmetry of the order parameter equations and the corresponding Poisson manifold governs the system approximately and we call this approximation the expectation assumption.

For general coupled oscillators, this assumption only works in the limit *N* → ∞, which is suitable for a large system. This is the traditional continuous model we used to describe a large system, independent of the dynamic and distribution of the system. When phases of the oscillators are chosen from a Poisson distribution, one gets that the assumption also works in vicinity of synchrony, with 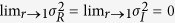 in Eq. (36), even for a small system. Adding more information about the system we can get more broader conditions for this assumption.

Of course, these two conditions are not the only ones for this assumption. In the following, we will consider more situations about order parameter equations and make the conditions of this assumption even broader. When the coupling function has some special terms and makes expectations of order parameters satisfy the same dynamical equations as Eq. (28), this assumption works even when a system is very small (contains a very small number of oscillators) and when oscillators in a system are far from the vicinity of synchrony.

#### The star Sakaguchi-Kuramoto model

In the following, let us take the star Sakaguchi-Kuramoto model as an example, which is a typical topology and model in grasping the essential properties of heterogeneous networks and synchronization process, as


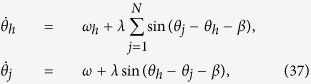


where 1 ≤ *j* ≤ *N*, *ω*_*h*_, *θ*_*h*_ and *ω*_*j*_, *θ*_*j*_ are the natural frequency and the phase of hub and leaf nodes respectively, *λ* is the coupling strength and *β* is the phase shift. By introducing the phase difference *φ*_*j*_ = *θ*_*h*_ − *θ*_*j*_, the dynamical equation can be transformed into


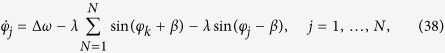


where Δ*ω* = *ω*_*h*_ − *ω*_*j*_ is the natural-frequency difference between hub and leaf nodes.

By introducing the order parameter 
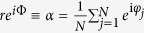
, we can rewrite the dynamics (38) as


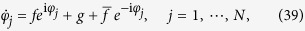


where 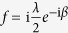, *g* = Δ*ω* − *λ* *Nr* sin (Φ + *β*). Hence Eq. (39) fall in the framework that we discussed above, with the first three nonzero Fourier coefficients of the coupling function. By applying our approach, the dynamical equations of order parameters can be obtained as


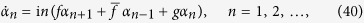


which can not be used directly to describe the finite system.

In a traditional method, for a large finite system we can set a hypothetical system as


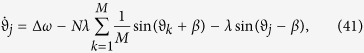


where 1 ≤ *j* ≤ *M*, *ϑ*_*j*_ is the hypothetical phase oscillator and *N* is considered as a parameter in this system. Defining the order parameter for this system as 
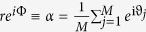
, Eq. (41) becomes


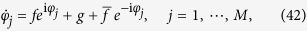


where 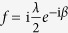, *g* = Δ*ω* − *λ* *Nr* sin (Φ + *β*). By taking the limit of infinitely many oscillators for this hypothetical system *M* → ∞, we can get a continuous model for Eq. (42) as


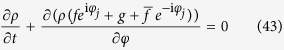


where *ρ*(*φ*, *t*) is the phase density of the infinite hypothetical system. There are many analytical methods on this continuous model, and traditionally we assume that the results getting from this model can be used to describe the large finite system, taken into account some perturbations around 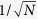.

In our approach, this traditional method is just about the first condition of the expectation assumption, i.e., the large size of the system, with no more information about distributions or dynamics of the system. The order parameter equations of the hypothetical system Eq. (42) read


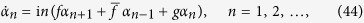


which are exactly the same as Eq. (40). In the the limit of infinitely many oscillators, *M* → ∞, the dynamics of Eq. (44) describes the behaviors of the hypothetical system exactly. Using the continuity equation, the traditional method for large systems can also be considered as using the order parameter equations to describe the behaviors of the finite system, under the first condition of the expectation assumption.

However, when more information about distributions and dynamics of coupled oscillators one can obtain more results for specific systems. Expectation assumption implies that every trajectory with initial states chosen randomly from a probability density in Poisson manifold can be described by the corresponding invariant manifold approximately. Therefore one gets an ensemble of these trajectories, we can expect that the ensemble average over all these trajectories will also follow the dynamics on the invariant manifold approximately. On the other hand, for some systems, even if we can get the dynamics of the ensemble average of trajectories directly on Poisson manifold, one can expect the trajectories in this ensemble is approximately described by the corresponding invariant manifold in the sense of probability and the expectation assumption works.

According to our above viewpoints for the described by dynamics Eq. (41), we have the dynamical equations of the expectation of order parameters as


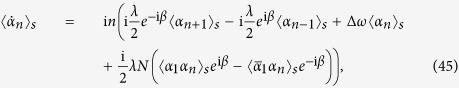


where *n* = 1, 2, … and 〈·〉_*s*_ means the expectation. By setting


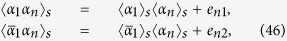


for *n* = 1, 2, …. Expressions (46) imply the condition that the expectation of multiplications of two parameters at the left-hand sides can be decomposed into the product of their individual expectations, i.e., if the modulus of the terms *e*_*n*1_ and *e*_*n*2_ are much smaller than the dominating term 〈*α*_*n*−1_〉_*s*_ in Eq. (45). In this case we can ignore *e*_*n*1,*n*2_ and get the dynamical equations as


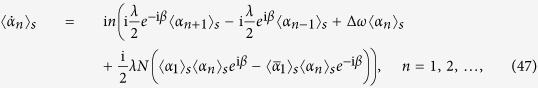


which is the same order parameter equations as Eq. (40) for the finite system and Eq. (44) for the hypothetical system.

When the states of the system are chosen randomly from a probability density in Poisson manifold, by taking the expectation among a Poisson distribution, we have


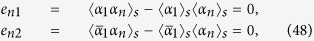


which means that with an expectation among the Poisson distribution, the expectation of order parameters satisfy the same dynamical equations with the finite system, according to our above viewpoints, independent of the size and states of the system. As Poisson manifold is an invariant manifold for this system, the expectations of order parameters will evolve on this manifold. We can get the expectation of trajectories directly through the dynamical equations and get the properties of the system approximately in the probability sense, even for a small system and when the system is far from synchrony.

**Figure 1:**
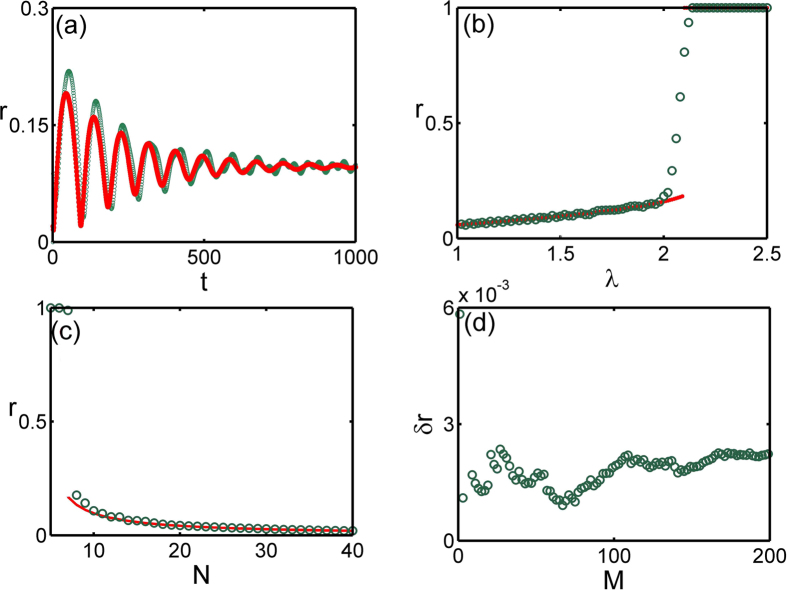
(**a**–**c**) Order parameter getting from numerical simulations (ensemble average with light blue circles) and theoretical approximated OA ansatz (red line), with *N* = 10, Δ*ω* = 9, *β* = −0.1*π*, *λ* = 1.5, *M* = 200 and varying time *t* in (**a**), *t* = 50, *N* = 10, *M* = 200, Δ*ω* = 9, *β* = −0.1*π* and varying coupling strength *λ* in (**b**), *t* = 50, *λ* = 1.5, *M* = 200, Δ*ω* = 9, *β* = −0.1*π* and varying size of the system *N* in (**c**). (**d**) The difference between the ensemble average and the theoretical result with light blue circles with *t* = 50, *N* = 10, *λ* = 1.5, Δ*ω* = 9, *β* = −0.1*π* and varying size of the ensemble *M*.

We can check this statement through the differences between the ensemble average of trajectories in numerical simulations and the theoretical order parameter’s expectation. In Fig. 1, we show these differences with different time *t* in (*a*), different coupling strengths *λ* in (*b*), different size of the system *N* in (*c*) and different size of the ensemble *M* in (*d*). It is clear that the expectation assumption works and the theoretical expectation of order parameters on Poisson manifold can be used to describe the behaviors of the finite system and cases of dynamical states far from or near synchrony.

With this expectation assumption, the order parameter equation in the bounded space as |*z*| ≤ 1 describes the dynamics of this coupled oscillator system. Every stationary state of a phase-oscillator system has its counterpart in the space of an order parameter. In this two-dimensional order-parameter space, it is easy to analyze the existence and stability conditions of these states, and also their basins of attraction^17^.

The Poisson manifold is useful to discuss behaviors of systems with both infinite and finite sizes. The restriction above is that the coupling function contains only the first two harmonic terms. It is worthwhile to note that, in refs 8 and 9, the authors developed the so-called Watanabe-Strogatz (WS) theory to provide an exact description of globally coupled identical phase oscillators with sinusoidal coupling for any number of oscillators in terms of the effective three-dimensional dynamics. This is a powerful tool, and one can use it to get similar results as we get here for finite oscillators, which shows approximate two-dimensional collective behaviors^13^, based on the evolution of a single trajectory. On the other hand, in the frame of the order parameter approach we have discussed above, we get a precise way to get and understand this approximate two-dimensional collective behaviors in the ensemble sense. In the following section, we will use the order parameter equations to further analyze systems with complex coupling functions, which is obviously out of the scope of the OA anstaz or the WS theory. We will find that the OA manifold can also be used to describe these systems approximately, and we can get some analytical results in the frame of order parameter equations.

#### Complex coupling function

In the above sections, for systems with coupling functions with only first three terms as


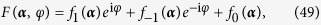


we have the dynamical equations of order parameters {*α*_*n*_} as


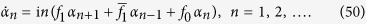


On the invariant manifold 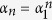, all these equations are reduced to a single one


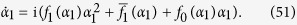


In general situations, the coupling function cannot be simply truncated to only the first two terms. It is thus important to consider the order parameter approach for more complicated cases.

#### Higher order coupling only

Firstly, let us consider a coupling function with only higher order Fourier coefficients like


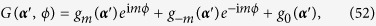


where *m* > 1 is an integer and ***α***′ are the order parameters of the system. Note that Eq. (52) can be regarded as a transformation of Eq. (49) with *φ* = *mϕ* and *f*_*j*_ = *g*_*j*·*m*_.

Let us take the case *m* = 2 as an example^18^. In this case, only *f*_±2_ and *f*_0_ among the Fourier components of the coupling function are nonzero, i.e.,


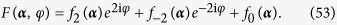


By using our order-parameter-analysis approach, when *m* = 2, it is not hard to get the equations of order parameters as


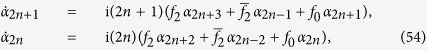


where *n* = 1, 2, ….

It is obvious that the order parameter equation (54) can be divided into two independent parts, the odd part and the even part. For the even part,


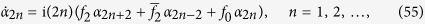


supposing *α*_2*n*_ = *H*(*α*_2_, 2*n*), through the similar approach we discussed in the first section, we have


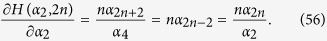


From the third equality, the function *H*(*α*_2_, 2*n*) can be got through


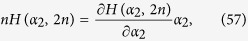


as 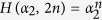. In fact, by setting 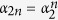, one can reduce the equations to a single one as


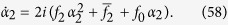


This is quite similar to the systems with coupling functions of only the first harmonic terms.

On the other hand, for the odd part, one has


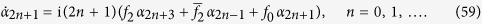


one can find that the OA analysis becomes different. Supposing *α*_2*n*+1_ = *H*(*α*_1_, 2*n* + 1), we have


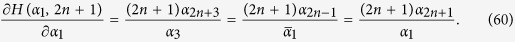


From the third equality, the function *H*(*α*_1_, 2*n* + 1) can be got through


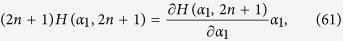


as 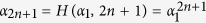. However, the second equality of Eq. (60) is not consistent with this solution as


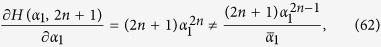


which means that the solution with the form *α*_2*n*+1_ = *H*(*α*_1_, 2*n* + 1) does not exist in general, apart from the special case 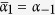.

The above inconsistence can also be considered as follows. If we just substitute the relation 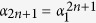 into the odd part, two equations other than one will be reduced to


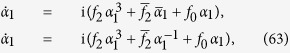


where the first equation is got from *n* = 0 in the odd part, while the second equation is obtained from all the others *n* > 0. If the relation is justified to describe the odd part, the consistence condition of these two equations in Eq. (63) requires 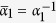. This condition implies |*α*_1_| = 1, which means that there isn’t a general solution of *α*_2*n*+1_, except in the vicinity of synchrony. Another special choice that satisfies the consistence condition is the trivial case as *α*_2*n*+1_ = *H*(*α*_1_, 2*n* + 1) = 0.

By putting the even and odd parts together, we can obtain the special solution as *α*_2*n*+1_ = 0, 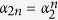, *n* ≥ 0. This invariant manifold separates the relation 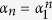 into two parts, where the part 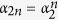 conserves the relation, and the other part *α*_2*n*+1_ = 0 can be regarded as a special choice of the relation. Even if the system is in the states as |*α*_2*n*_| = |*α*_2_|^*n*^ = 1, we still have *α*_1_ = 0, representing the states of cluster synchrony as discussed in ref. 18. The manifold with *α*_2*n*+1_ = 0, 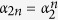, *n* ≥ 0 can be regarded as a sub-manifold of Poisson manifold.

#### Mixture of higher order couplings and the Dominating-term assumption

Consider a system with a coupling function which has the first five nonzero Fourier coefficients, as


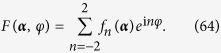


The corresponding equations of order parameters read now


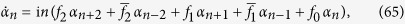


with 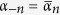, *n* = 1, 2, …. In this case, if we suppose 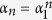, then two different dynamical equations will be obtained


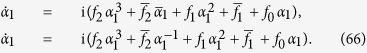


Hence we will have 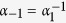 as the requirement of consistency of these two equations. The relation 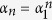, *n* > 0 is broken by the conjugate terms and there is no way in finding out a separated solution as we did for Eq. (54). The only solutions with the relation 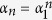 for this system seems to be either 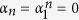 or |*α*_*n*_| = |*α*_1_|^*n*^ = 1. None of them are expected for our analysis, unless in the vicinity of synchronous states with |*α*_1_| = 1 or in the vicinity of incoherent states with *α*_1_ = 0.

On the other hand, supposing an function *α*_*n*_ = *G*(*α*_1_, *n*) and substituting it into the dynamical equation (65) we have


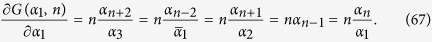


From the last equality we have the solution as


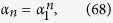


and except the second equality which involves the conjugate term 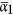, all the other equalities are satisfied by this relation. If the second equality can be satisfied approximately, as


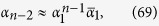


the relation 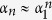 can be seen as a good approximation for this system. The consistence condition for these two approximations is


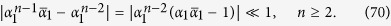


It is clear that this condition can be satisfied in the vicinity of synchronization states and incoherent states. In general, if we choose the initial conditions as 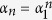, and along the evolution of Eq. (65), the differences between higher terms *α*_*n*_ and 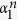 can keep small enough, the consistence condition can be satisfied naturally.

Through the analysis of the function *α*_*n*_ = *G*(*α*_1_, *n*), the dynamics on this approximated manifold are governed by the dynamical equation of the first order parameter as


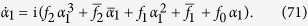


The second equation in Eq. (66) is ignored in this approach.

In the view of Eq. (66), we can see that the first equation is got from the dynamics of the first order parameter, and the second equation is got from the higher-order order parameters. For the relation of approximated OA ansatz, 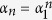, the higher terms are much smaller than the first one, and the first equation can be seen as the dominating equation, and the term can be regarded as the main term of the dynamics Eq. (65). From now on we call this approach *dominating-term assumption*. We will further show that this approximation is pretty efficient in dealing with systems with complicated coupling functions.

Let us take the system described by Eq. (38) as an example. By considering higher terms, we have the dynamical equations


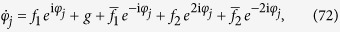


where 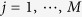 and 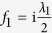, 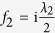, *g* = Δ*ω* − *λ*_0_*r* sin (Φ) with *re*^iΦ^ = *α*_1_. By introducing the dominating-term assumption, the dynamical equation for *α*_1_ reads


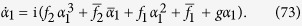


If the dominating-term assumption works, Eq. (73) can be used to study the collective behaviors of the coupled oscillators, which can be regarded as a sort of approximate low-dimensional behaviors.

**Figure 2:**
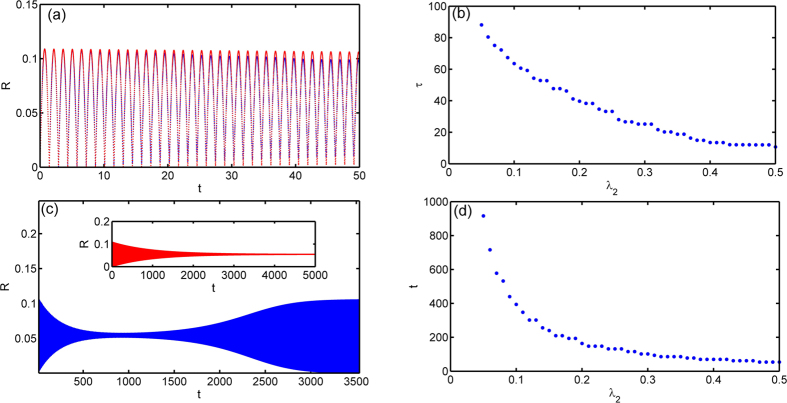
(**a**) Numerical results about *R*_*s*_ with different initial order parameters, as *α*(0) = 0.1(red) and *α*(0) = 0.4(blue), with *λ*_0_ = 7.135, *λ*_1_ = 0.5, *λ*_2_ = 0.1. (**b**) The separation of basins of attraction of incoherent state and synchronous state, calculated from numerical simulations of oscillators(red) or Eq. (73) (blue), with *λ*_0_ = *Nλ*, *λ*_1_ = 0.5, *λ*_2_ = 0.1, *N* = 3. The other parameters are Δ*ω* = 5, *dt* = 0.01, *M* = 20000.

The validity of this dominating-term assumption can be checked via numerical simulations. When initial states of the system are chosen from a Poisson distribution, we can make a comparison of the evolutions between the order parameter from dynamical equations of coupled oscillators *α*_*s*_ and the theoretical one from the approximate manifold *α*_*o*_. We find that the dominating-term assumption works for systems with higher harmonics in a finite time scale *τ*, and *τ* → ∞ when the coupling strength of higher harmonics *λ*_2_ → 0. In Fig. 2(a) the modulus of the order parameter *R* = |*α*| is plotted. It can be found that the theoretical results with dominating-term approximation coincides very well with numerical results in over 50 time unites. We can measure the time scale *τ* that the dominating-term assumption works through the relative error, defined as 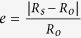, as shown in Fig. 2(b), which depends on *λ*_2_. It is clear that *τ* can be arbitrarily large when the coupling strength *λ*_2_ is small enough, which means that the system shows an approximate low-dimensional behaviors in the presence of small higher harmonics couplings and the dominating-term assumption is a natural and effective means in revealing these behaviors.

**Figure 3:**
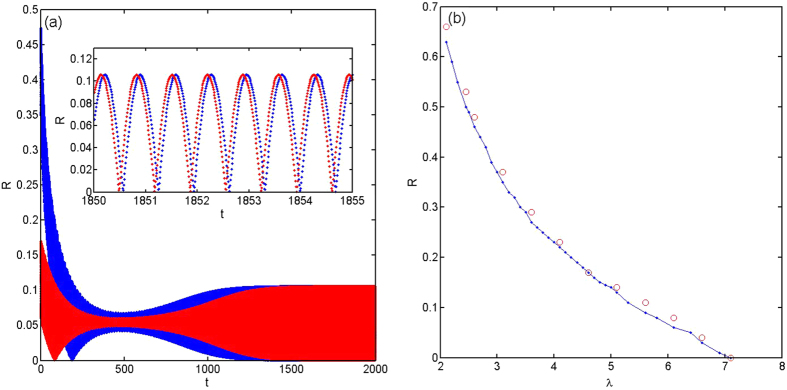
(**a**) Numerical results about *R*_*s*_ (blue) and *R*_*o*_ (red) with *λ*_2_ = 0.1. (**b**) The time scale *τ* for dominating-term assumption defined as 
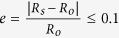
 with different *λ*_2_. (**c**) Numerical results about *R*_*s*_ (blue) and *R*_*o*_ (red) with *λ*_2_ = 0.1. (**b**) The time scale defined as the time of a trajectory evolving into the vicinity of a attractor (amplitude of *R* stay stale and around the attractor for a span) with different *λ*_2_. The other parameters are *λ*_0_ = 7.135, *λ*_1_ = 0.5, Δ*ω* = 5, *dt* = 0.01, *M* = 20000 and the initial order parameter is *α*(0) = 0.

It should be pointed out that as 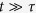, the OA manifold and the corresponding order parameter equation can still be applicable to describe the low-dimensional collective behaviors in the qualitative sense. This validity is because the system of oscillators can still evolve to the vicinity of the corresponding attractor of Eq. (73) (with the same initial state) in the long time range, even beyond the time scale for the dominating-term assumption, as shown in Fig. 2(c). Moreover, the systems in the same basins of a attractor of Eq. (73) will evolve to the vicinity of the same attractor, and when they evolve out of the vicinity of the attractor they will evolve to the same stable state of the system as shown in Fig. 3(a). This behavior indicates that the OA manifold is so robust in influencing the behaviors of the oscillators, and the mechanism behind this is still an open question. The time scale for the attraction is measured as the moment when the trajectory evolve into the vicinity of a attractor (the amplitude of the order parameter stay stable and around the attractor for a span), as shown in Fig. 2(d), which is a rather long time scale, about ten times of *τ* and also goes to infinity in the limit *λ*_2_ → 0. We can also use this influence of the OA manifold, as Eq. (73), to determine the basins of attractions of the incoherent state and the synchronous state, as shown in Fig. 3(b), which has high accuracy apparently.

The above results indicate we can see that the dominating-term assumption works pretty well in the presence of high-order coupling terms, and this substantially extends the scope of the understanding of the OA manifold. With the approximated order parameter equation (73), we can keep going ahead to find properties of collective behaviors of the system, which will be discussed in detail in another paper.

## Discussion

In this paper, we focused on theoretical descriptions of low-dimensional order-parameter dynamics of coupled phase oscillators. We have derived a closed form of dynamical equations of order parameters, from which we find the Poisson kernel as an invariant manifold and the well-known OA ansatz. Different from traditional Ott-Antonsen ansatz and Watanabe-Strogatz’s approach, our approach is suitable for systems of both finite and infinite, identical and nonidentical oscillators. In our approach, the scope of OA ansatz is determined as two parts, i.e., the limit of infinitely many oscillators and the condition that only the first three Fourier coefficients of the coupling strength are nonzero.

By using our order parameter analysis, we also discussed two cases that go beyond the scope of OA ansatz, i.e., finite-size systems and coupled oscillator systems with more complicated coupling functions. We have discussed the reasons why OA ansatz cannot be used directly to these systems and further developed approximation methods to deal with these difficulties. We developed two approaches, namely the expectation assumption and dominating-term assumption. It is shown that these schemes work pretty well, and their validity has been checked by numerical simulations.

In this paper, we have developed the order parameter analysis, with which we get the low-dimensional behaviors of coupled phase oscillators, such as OA ansatz, the expectation assumption and the dominating-term assumption. However, to fully understand the low-dimensional collective behaviors we need a more comprehensive understanding of Poisson manifold and its relation with the dynamics of oscillator systems, which is still an open question. We believe that the order parameter analysis should be a powerful tool in helping to reveal the mechanism of low-dimensional collective behaviors.

## Additional Information

**How to cite this article**: Gao, J. *et al.* Order parameter analysis for low-dimensional behaviors of coupled phase-oscillators. *Sci. Rep.* **6**, 30184; doi: 10.1038/srep30184 (2016).
